# Unveiling the Wing Shape Variation in Northern Altiplano Ecosystems: The Example of the Butterfly *Phulia nymphula* Using Geometric Morphometrics

**DOI:** 10.3390/ani14192758

**Published:** 2024-09-24

**Authors:** Thania Acuña-Valenzuela, Jordan Hernández-Martelo, Manuel J. Suazo, Isabel A. Lobos, Alejandro Piñeiro-González, Amado Villalobos-Leiva, Franco Cruz-Jofré, Raquel Hernández-P, Margarita Correa, Hugo A. Benítez

**Affiliations:** 1 Laboratorio de Ecología y Morfometría Evolutiva, Centro de Investigación de Estudios Avanzados del Maule, Universidad Católica del Maule, Talca 3466706, Chile; u20201186727@usco.edu.co (T.A.-V.); jordan.hernandez.01@alumnos.ucm.cl (J.H.-M.); isabelobos3@gmail.com (I.A.L.); andresale96@gmail.com (A.P.-G.); avillaleiv@gmail.com (A.V.-L.); mcorreag@ucm.cl (M.C.); 2 Semillero de Investigación AGROCON, Facultad de Ciencias Exactas y Naturales, Universidad Surcolombiana, Avenida Pastrana Borrero Carrera 1, Barrio Santa Inés, Neiva 410001, Huila, Colombia; 3 Cape Horn International Center (CHIC), Centro Universitario Cabo de Hornos, Universidad de Magallanes, Puerto Williams 6350000, Chile; 4 Programa de Doctorado en Salud Ecosistémica, Centro de Investigación de Estudios Avanzados del Maule, Universidad Católica del Maule, Talca 3460000, Chile; 5 Instituto de Alta Investigación, Universidad de Tarapacá, Casilla 7D, Arica 1000000, Chile; suazo.mj@gmail.com; 6 Research Ring in Pest Insects and Climate Change (PIC2), Santiago 8320000, Chile; 7 Escuela de Medicina Veterinaria, Facultad de Recursos Naturales y Medicina Veterinaria, Universidad Santo Tomás, Ejercito Libertador 146, Santiago 8370003, Chile; francocruzjo@santotomas.cl; 8 Departamento de Ecología Evolutiva, Instituto de Ecología, Universidad Nacional Autónoma de México, Ciudad de México 04510, México; raquel.hernandez@ecologia.unam.mx

**Keywords:** Pieridae, shape analysis, Altiplano, extreme environment, butterflies, morphometrics

## Abstract

**Simple Summary:**

The Andean Altiplano, known for its extreme weather and high biodiversity, is an ideal place to study how insects adapt to their environment. This research focuses on the butterfly species *Phulia nymphula*, which is common in the high-altitudinal Andes Mountains, to identify how their wing shapes vary across six locations in the Northern Chilean Altiplano. By analyzing the wings of 77 butterflies, the study found significant differences in wing shape, likely due to local environmental conditions. These differences suggest that the butterflies have adapted to their specific habitats. The findings showed how the wing shape differentiate between localities across the Northern Altiplano and provide insights into how high-altitude species evolve and adapt through changes in their morphology, highlighting the role of ecological and evolutionary processes in shaping biodiversity in extreme environments.

**Abstract:**

The Andean Altiplano, characterized by its extreme climatic conditions and high levels of biodiversity, provides a unique environment for studying ecological and evolutionary adaptations in insect morphology. Butterflies, due their large wing surface compared to body surface, and wide distribution among a geographical area given the flight capabilities provided by their wings, constitute a good biological model to study morphological adaptations following extreme weathers. This study focuses on *Phulia nymphula*, a butterfly species widely distributed in the Andes, to evaluate wing shape variation across six localities in the Northern Chilean Altiplano. The geometric morphometrics analysis of 77 specimens from six locations from the Chilean Altiplano (Caquena, Sorapata Lake, Chungará, Casiri Macho Lake, Surire Salt Flat, and Visviri) revealed significant differences in wing shape among populations. According to the presented results, variations are likely influenced by local environmental conditions and selective pressures, suggesting specific adaptations to the microhabitats of the Altiplano. The first three principal components represented 60.92% of the total wing shape variation. The detected morphological differences indicate adaptive divergence among populations, reflecting evolutionary responses to the extreme and fragmented conditions of the Altiplano. This study gives insights into the understanding of how high-altitude species can diversify and adapt through morphological variation, providing evidence of ecological and evolutionary processes shaping biodiversity in extreme environments.

## 1. Introduction

The Andean Altiplano, located at an elevation between 3700 and 4600 m.a.s.l., encompasses various hydrographic basins delineated by two mountain ranges of the Central Andes, with a system of wetlands and lakes separated by fragmented areas [1]. The Chilean Altiplano is a narrow strip from approximately 17°30′ S to 24° S, with an approximate altitude of 4000 m above sea level, featuring mainly steppe vegetation due to its dry and cold climate [2,3]. Altiplano weather is characterized by a low atmospheric pressure, low air density, high radiation, and minimal atmospheric humidity, where regional winds from the east transport moisture over the Andes from the Amazon basin [2].

These extreme climatic conditions contribute to a highly endemic Andean biodiversity, with species adapted to the different habitats [4,5,6]. Regarding the entomofauna of the Altiplano, the most outstanding groups are the Coleoptera and Lepidoptera. Andean butterflies are characterized by a high mobility between latitudinal and altitudinal gradients [7] and with a flight period associated with temperature limitations in different seasons along this gradient [8]. Even though the diversity of butterflies as a taxonomic group has been little studied in Chile [9], a total of five families have been reported for the country, with four of them (Hesperiidae, Lycaenidae, Nymphalidae, and Pieridae) being present in the Altiplano [7,10,11,12,13]. The taxonomy of the family Pieridae has been studied in detail [14,15,16], and at least 13 species belonging to the genera *Phulia*, *Infraphulia*, *Pierphulia*, and *Piercolias* are considered high-Andean pierines (Shapiro et al., 2007). A systematic study by Pyrcz [17] combined all of these species under the genus *Phulia*, with *Phulia nymphula* being one of the most representative species, with a wide distribution in the Andes in an altitude between 2300 and 5000 m.a.s.l. It prefers low vegetation, such as grasses and areas with low shrubs, and is directly associated with swamps, although to a lesser extent than other *Phulia* species [18,19]. Morphologically, it is characterized by a yellowish dorsal side in both sexes, with a black stripe in the discal cell and spots on the outer and submarginal edges, which are more noticeable in females [13]. In addition to coloration, wing morphology also differs between sexes, encompassing aspects such as size, shape, and venation. All these are relevant for ecological and adaptive studies of the species in this group [20,21,22,23,24].

Butterflies have the largest wing surface area relative to body mass among insects [25,26]. Environmental pressures and geographic distances are responsible for creating geographic microenvironments, and therefore modifying their associated flora and fauna. Insect species have the ability to adapt to specific environments over time, with variations in wing morphology directly reflecting this ability in flying species [26]. Understanding the interaction between complex phenotypes and environmental factors, as well as genetic and/or biomechanical factors, allows us to relate morphological variation to functional–ecological adaptations with environmental variation [12,27,28,29].

The development of multivariate statistics and tools such as geometric morphometrics (GM) has allowed the study of biological shape by evaluating quantitatively the morphological variations between study groups [30,31,32,33]. GM enables the analysis of the shape of organisms or specific structures by considering geometric shape, focusing on the geometric properties that persist once the effects of scale, rotation, and translation are removed from an object [34]. This approach offers a more thorough biological interpretation than mere qualitative comparison [35], with robust analyses that allow the visual representation of morphometric variation within and between populations [36,37]. Several wing studies illustrate this; for example, Lemic et al. [38] reported significant differences in the wing shape of the box tree moth (*Cydalima perspectalis*) between terrestrial and coastal populations in Croatia, as well as a subtle sexual dimorphism, suggesting that this morphological variability may have implications for the species’ invasive capacity and spread. Similarly, Cardenas-Muñoz et al. [39] studied butterflies of the family Nymphalidae in the Chocó rainforest of Ecuador, finding that butterflies in different strata show distinct morphological adaptations, with those in the canopy having characteristics associated with fast flight, while those in the understory exhibit adaptations for slow gliding.

Since Lepidoptera are one of the most numerously represented insect groups in the Altiplano and *Phulia nymphula* one of the most distributed butterflies in the Andes, the possible presence of morphological adaptation of a butterfly species in the Altiplano weather was studied. Considering the qualities of butterflies and the extreme environment of the Altiplano as optimal for analyzing morphological changes, the present study aims to evaluate wing shape variation in two basins of the Chilean Altiplano of the species *Phulia nymphula* using geometric morphometrics tools to understand the morphological adaptation to specific climatic conditions.

## 2. Materials and Methods

### 2.1. Sampling and Data Acquisition

**Figure 1:**
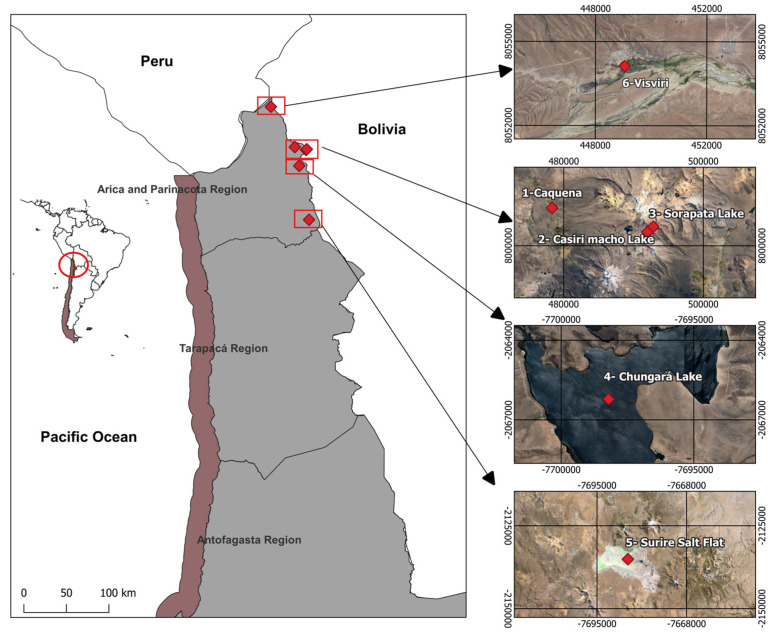
Map with the sampling locations used for this study: Caquena, Casiri Macho Lake, Sorapata Lake, Chungará, Surire Salt Flat, and Visviri.

A total of 76 specimens of *Phulia nymphula* were collected throughout the Northen Chilean Altiplano, specifically from six localities: (1) Caquena, with shrubland areas on low slopes; (2) Casiri Macho Lake and (3) Sorapata Lake, both lakes with wetland vegetation near bodies of water; (4) Chungará, characterized by bofedales (high Andean wetlands) and shrubland areas on hillside slopes; (5) Surire Salt Flat, high Andean wetland with herbaceous vegetation near bodies of water; and (6) Visviri, where bofedales with herbaceous vegetation are evident (Figure 1).

An asynchronous sample collection was conducted using an entomological net during April 2018, March, and May 2019; it is important to mention that not all localities were sampled in the same year. The taxonomic classification of the individuals was verified following the specific taxonomic keys of Peña and Ugarte [13]. Once collected, the butterflies were taken to the Laboratorio de Ecología y Morfometría Evolutiva at the Universidad Católica del Maule, Talca, Chile, for processing.

### 2.2. Geometric Morphometric Analyses

The right forewing of each individual sampled was removed and fixed on a slide by means of the wing extension. To obtain images with a reference scale, the wings were arranged on scale paper. Photographs were captured using a Nikon D7500 (Nikon Sales, Bangkok, Thailand) camera with a 100 mm macro lens and processed in TPS format using tpsUtil32 v1.81. A total of 17 landmarks were established at vein intersections of the wing using tpsDig2 v2.31 software [40]. Samples showing wing damage were excluded from the analyses.

In order to determine the measurement error, the dataset was digitized twice, and using a Procrustes ANOVA, it was verified that the mean square (MS) values of the individuals were lower than the error [41]. After a generalized Procrustes analysis using the software MorphoJ v1.06a, the Cartesian coordinates resulting from the landmark placement were processed, thereby eliminating the elements of translation, rotation, and scale [34]. To graphically visualize the shape space, a principal component analysis (PCA) was performed using the covariance matrix of the individuals [42]. Similarly, to represent the average shape of individuals from each locality, an averaged covariance matrix was performed to extract the average shape of the different localities. In order to reduce the number of shape dimensions for visual examination among the different localities, a canonical variate analysis (CVA) was performed. It is important to note that this discriminant analysis aims to maximize the variation between groups by creating new shape axes [43]. To evaluate the statistical significance of the morphological variation between populations, a permutation test (10,000 permutations) was carried out using Mahalanobis distances (distances resulting from the CVA). Finally, in order to evaluate if whether size effects shape in these populations, a multivariate regression between shape (dependent variable) and centroid size (independent variable) and a violin plot of the distribution of size between localities were performed. A Procrustes ANOVA was performed using the locality as a classifier to identify if there was any statistical relationship between size and shape variables. All analyses were performed using the MorphoJ 1.08.02 [44] software and R packages, geomorph, and ggplot2 [45,46].

### 2.3. Wing Shape and Environment

Information on wind speed was extracted from models developed by the Chilean government for wind energy exploration https://eolico.minenergia.cl/inicio (Accessed on 12 September 2024). The data used come from the RECON 1980–2017 model, which contains 333,116 wind speed records (m/s), with 180,440 records covering the period from 6:00 to 18:00 h. These measurements were estimated at a height of 5.5 m above the ground and span a period of 37 years (spatial resolution: 1 km) [47]. We calculated the average and maximum wind speeds for each location and categorized them into three groups based on maximum wind speeds recorded between 6:00 and 18:00 h: LOW (<20 m/s), MEDIUM (20–25 m/s), and HIGH (>25 m/s). Additionally, wind force was interpreted using the Beaufort scale, as outlined by the Chilean Army.

A PERMANOVA analysis was conducted using the software PAST version 3.26 [48] to assess differences in wing shape, categorized by average wind speed (LOW, MEDIUM, and HIGH). The principal component scores obtained for each individual were used as variables related to wing morphology.

## 3. Results

**Figure 2:**
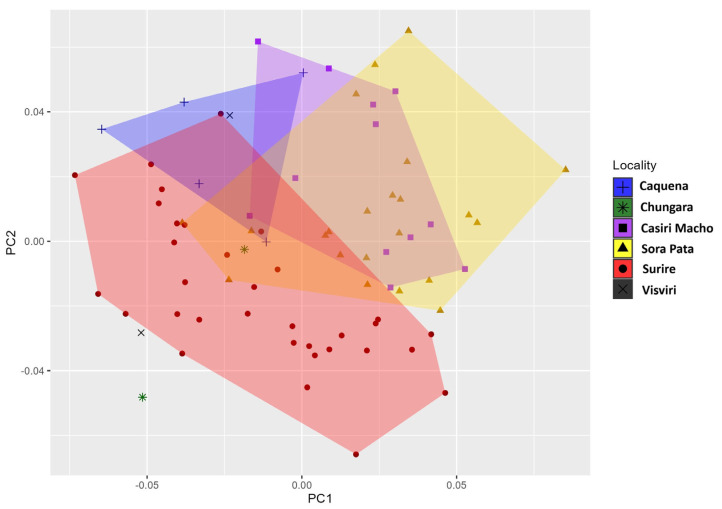
Principal component analysis of *Phulia nymphula* Northern Altiplano localities. Blue: Caquena, yellow: Sorapata Lake, green: Chungará, purple: Casiri Macho Lake, black: Surire Salt Flat, and brown: Visviri.

The first three principal components (PCs) of the PCA distribution of shape for the six study locations explain 57.823% (PC1 + PC2 + PC3: 25.385% + 17.262% + 15.176%) of the total shape variation, providing an approximation of the total amount of variation, since the other PC components represent no more than 42.177% of the total variation (Figure 2).

**Figure 3:**
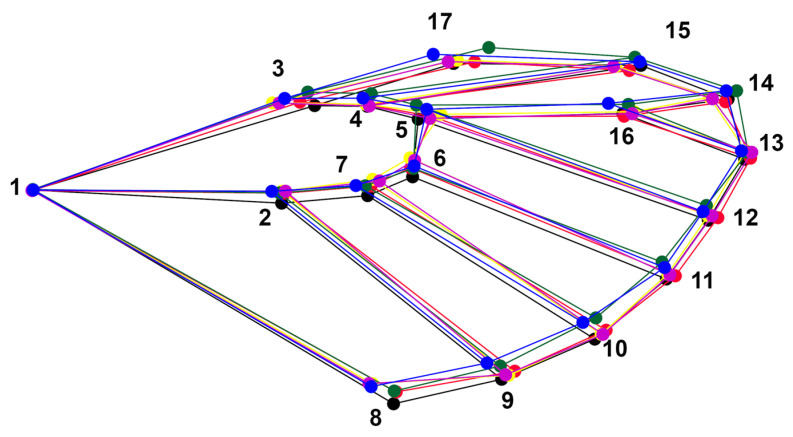
Superposition of the average shape (PCA) of *Phulia nymphula* represented by a colored wireframe between localities. Blue: Caquena, yellow: Sorapata Lake, green: Chungará, purple: Casiri Macho Lake, red: Surire Salt Flat, and black: Visviri.

The superimposition of the mean shapes of every population shows that the differences are accentuated at the following landmarks. At LM#5, the locations showed significant displacement from each other, with Chungará (represented by green) a bit further to the left, followed by Visviri and the Surire Salt Flat, which is overlapping with Caquena, Sorapata Lake, and Casiri Macho Lake. LM# 1, 2, 3, 4, 6, and 7 also show displacement in the populations that are overlapping, and it is consistently observed that the locality of Chungará tends to remain more compact and smaller compared to the others. This is further confirmed when analyzing the displacements of points 9 to 13, where all populations tended to the right (indicating a wider wing shape) compared to the average shape of the individuals from Chungará (Figure 3).

**Figure 4:**
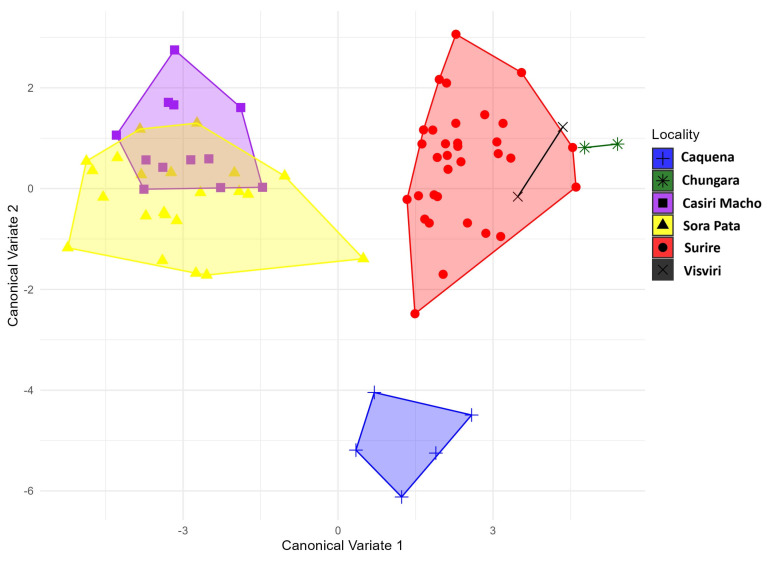
Canonical Variate analysis of *Phulia nymphula* wing shape between 6 localities at Northen Altiplano. Blue: Caquena, yellow: Sorapata Lake, green: Chungará, purple: Casiri Macho Lake, red: Surire Salt Flat, and black: Visviri.

The CVA maximized the variance to create new axes and provide a discrimination graph, which confirmed that the *P. nymphula* of different localities of are clearly separated in their shape (Figure 4). A permutation test based on the Mahalanobis distances between populations showed that populations differ significantly in wing shape (Figure 4, Table 1).

**Figure 5:**
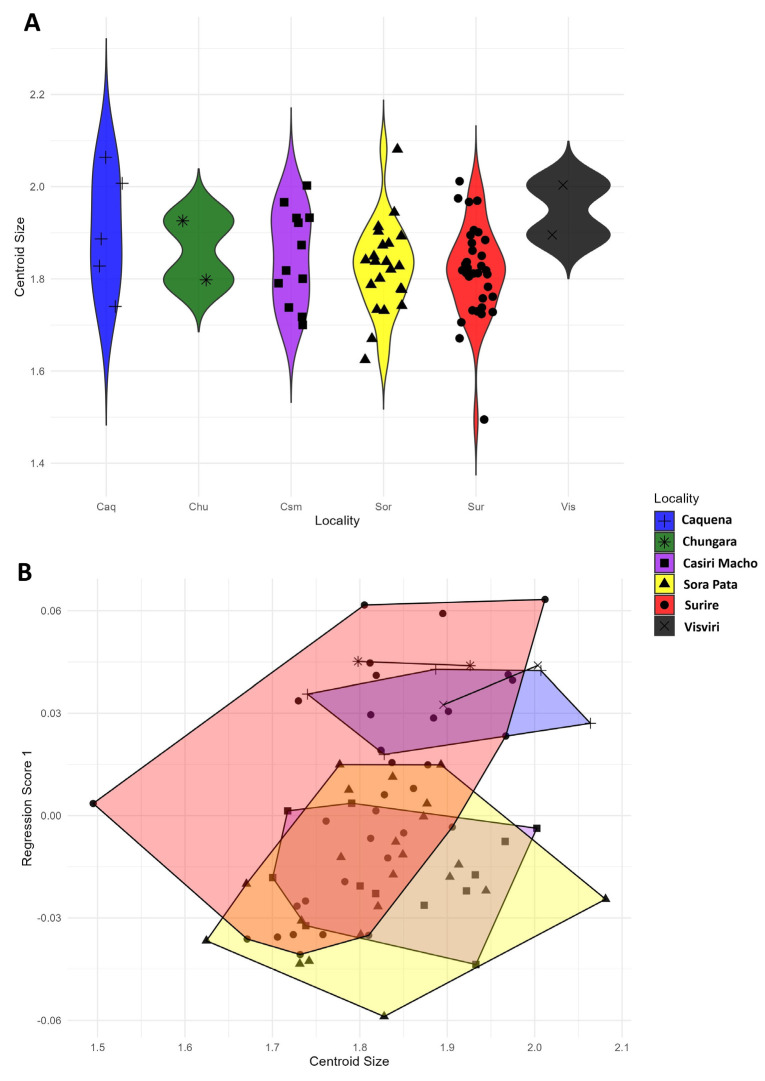
(**A**): Violin plot of the distribution of centroid sizes between localities. (**B**): Multivariate regression of the wing shape of *P. nymphula* between different localities. Blue: Caquena, yellow: Sorapata Lake, green: Chungará, purple: Casiri Macho Lake, red: Surire Salt Flat, and black: Visviri.

Finally, a comparison between the centroid sizes of the multiple localities was performed using a violin plot of the centroid size distribution; a very similar C. size distribution can be observed (Figure 5A), In order to test whether any of the weak centroid size differences between populations affect our results, multivariate regression showed a clear similarity of shape by the influence of the centroid size of *P. nymphula* with 2.65% of allometric influence (Figure 5B). The Procrustes ANOVA confirmed a non-significant relationship of the centroid size between localities (F: 1.33, *p*-value: 0.2603).

Table 2 presents the average and maximum wind speeds for each location. It can be observed that the locations of Sorapata Lake and Casiri Macho Lake have the highest average wind speeds (between 6 and 8 m/s), with maximum speeds approaching 30 m/s. The lowest values were observed in Caquena, Visviri, and Salar de Surire, ranging between 4 and 5 m/s, with the maximum speeds also being lower, between 17 and 22 m/s.

The one-way PERMANOVA analysis showed a significant effect of wind speed on wing shape (principal components) (F: 8.007; p-same: 0.0001; 9999 permutations), successfully differentiating the three categories (LOW; MEDIUM, and HIGH) in paired comparisons (*p* < 0.01; Bonferroni correction).

Supplementary Figure S1 shows that wind speed exhibits similar patterns across the years 2015 to 2017. Observing the range from 1980 to 2017 (Supplementary Figure S2), the months of austral winter and spring (June to November) exhibit the highest wind speeds (>20 m/s), with the variability in wind speed being similar across decades

## 4. Discussion

This study differentiated wing shape in butterfly *Phulia nymphula* localities from two basins of the Chilean Altiplano. Geometric morphometrics proved to be a powerful tool for characterizing and analyzing insect wing data. The variations presented by wings of the *Phulia nymphula* localities analyzed were likely influenced by local environmental conditions and selective pressures. The detected variations strongly suggest specific adaptations to the microhabitats of the Altiplano, with the first three principal components explaining close to 57.8% of the total wing shape variation.

Patterns of small phenotypic variations in wing morphology have shown significant implications for the fitness and flight capacity of butterflies in different environments [28,51]. As Dockx [52] indicated, studying the relationship between different migratory routes and behaviors with the wing morphology and conditions in migratory and resident monarch butterflies, *Danaus plexippus* (L.), specific morphological adaptations like these can confer selective advantages to populations in their local environment, which could influence their ability to survive and reproduce effectively [22,53].

Mikitová et al. [54], in a study conducted in the Western Carpathians, found that evident shape differences in males of the butterfly *Argynnis paphia* from five populations were correlated with environmental factors such as elevation, bedrock type, average annual temperature, and altitude. In the present study, our results showed that the geometry of the wing as a morphological trait separated each of the six localities in the Northen Altiplano. *P. nymphula* is characterized by a particular flight pattern that stays close to the ground with little elevation. This allows the species to expand its geographical range, but not over long distances. It typically rests with its wings extended, usually on clean soil without bushes. Our results showed that the overlapping of shape variations presented in the PCA reflect the shape characteristics needed to colonize the Altiplano’s environments, which share environmental conditions like strong wings and colder temperatures at night (down to a minimum of −15 °C).

Carneiro et al. [55] used wing shape as an environmental indicator, exemplified by *Euglossa cordata*. The authors employed morphology to detect environmental stress in populations from the Atlantic Forest, Savannah, and dry forest (Caatinga). They found that morphometric variability may be related to adaptations to the phytophysiognomic conditions of each locality.

In the case of the Altiplano, the common characteristics would be mainly associated with altitude and its effect on environmental variables (e.g., temperature). However, specific characteristics have been described in different areas of the Altiplano. For example, there is a gradient of higher precipitation in the northeast of the basin, which gradually decreases towards the south [56]. Additionally, changes have been observed in recent decades, leading to some areas in the Southern Altiplano (the Coipasa–Uyuni basin) experiencing lower humidity and higher temperatures [57]. These changes can be accentuated on a small scale depending on the conditions of each sub-catchment, which depend on its size and the presence of water bodies, glaciers, and topography affecting the habitat conditions (e.g., shelter, wind, and biological communities) for the species that live there. Our study found morphological differences at the basin scale, differentiating Caquena, Casiri Macho, and Sorapata from the Chungará localities. These basins present geographic barriers that may hinder the flow of individuals between them, in addition to having different environmental characteristics. Caquena and nearby localities are open areas with wind flow and are connected to the northeast with basins in Bolivia, which present suitable habitats for the reproduction of other butterflies above 5000 m.a.s.l. [10]. These basins have shown genetic differences among the organisms inhabiting them. In fish of the genus *Orestias*, cycles of isolation and connection between populations were detected [50], reaffirming a complex geological history in this area, which could be explained by large volcanic events during the Holocene [58,59] and recent climatic changes throughout the Altiplano basin [56].

The Surire Salt Flat basin, the most extensive in the study area (537 km^2^), exhibits the highest evaporation rates, potentially affecting resource availability. Although it is located at a high altitude (over 4000 m.a.s.l.), it remains lower in elevation compared to the northern basins, such as Caquena and Chungará, which are situated between 4600 and 5200 m.a.s.l. These characteristics could be key to the differentiation observed in *Phulia nymphula*; as in other species distributed in the area, significant differences in wing shape have been recorded along an elevational gradient, even in the absence of genetic differences, as in the example of the few differences found in the migratory butterfly *Vanessa carye*, at different elevational gradients on the Altiplano [12] and the multiple shape modifications that *Itylos titicaca*, a small lycenid, have from their habitat in *Oxychloe andina* (data to be published). Other research that has focused on exploring the mechanisms driving this interpopulation variation has identified direct relationships with developmental biology in the larvae of winged insects. Boggs and Niitepõld [60] examined the effects of larval dietary restriction on the morphology of butterflies of the species *Speyeria mormonia*, finding that this resulted in smaller individuals, with differences in body mass distribution and wing loading between sexes, highlighting the importance of resource allocation in nutrient acquisition under environmental stress. On the other hand, Starmer and Wolf [61], when analyzing the effect of temperature and larval density in 11 species (12 strains) of *Drosophila*, found that density and temperature during larval development influenced wing loading through general allometric relationships of body size and wing area.

The wind speeds recorded at each location generally show relatively low average values, corresponding to Beaufort Scale Forces 3 and 4, indicating wind speeds between 13 and 30 km/h. However, in locations with higher average wind speeds (Sorapata Lake and Casiri Macho Lake), the maximum recorded wind speeds exceed 100 km/h, categorizing them as Beaufort Scale Force 11 (Violent Storm). These locations are situated at elevations above 4800 m and are located on slopes near the edges of their respective basins; other locations (Caquena, Chungará, Visviri, and Surire Salt Flat) are characterized by lower elevations (4000 to 4600 m) and open areas in the lower parts of each basin. The geographical position and topography may influence wind exposure and speed on a small scale, as evidenced by the observed maximum wind speeds (>100 km/h). *P. nymphula* was observed flying during the day in windy conditions, similar to what has been recorded for other butterfly species in the same localities (e.g., *Vanessa carye*), However, these species would use microhabitats that protect them from the strong high-altitude winds [10]. Their flight is described as short, characterized by skimming flight with periods of resting on the ground with wings open [18,19,62]. This behavior contrasts with observations of smaller butterfly species, such as *Itylos titicaca or Phulia rosea*, which exhibit low mobility and avoid flying in windy conditions (Lobos, unpublished data). However, the threshold wind speed at which these butterflies exhibit activity was not assessed in this study.

The group of individuals inhabiting areas with high wind speeds (average 6 to 7 m/s and maximum wind speeds of 30 m/s) generally exhibited positive values in principal components 1 and 2, showing significant differences from the other two groups (LOW and MEDIUM wind environments). Individuals from Sorapata Lake and Casiri Macho Lake have, on average, lateromedially wider wings at the distal end compared to populations from Chungará and Surire Salt Flat (see Figure 4).

In biomechanical studies of wing shape, vortices generated at various wind speeds are typically larger at the leading edge and distal margin of the wing, which is also associated with changes in wing position [63]. Although we did not obtain experimental data on this aspect, the pattern found in our study is noteworthy. Longer wings were recorded in high-wind areas (Sorapata and Casiri Macho), which might seem counterintuitive given the increased stress on the wings. However, wing shape may be influenced by factors beyond environmental conditions, such as a low flight speed in species with aposematic signals (e.g., the genus *Danaus*) [63], which is not the case for *P. nymphula*, known for its less conspicuous coloration. Additionally, wing elongation patterns are observed in migratory species/populations [21]. Considering that *Phulia nymphula* is a specialist of high-altitude wetlands [7], its presence in this area suggests the fragmented distribution of its populations. The differences observed in our study likely represent local adaptations, possibly related to low mobility and limited connectivity between areas. This hypothesis should be further investigated with genetic markers and/or ecological studies (e.g., isotopes, niches, and movements).

## 5. Conclusions

Finally the findings presented so far in this research have implications for the understanding of the evolution [64,65,66] and ecology of butterflies in the Northern Altiplano. These results also provide insights for the future design of conservation strategies that need to take into account the morphological and genetic diversity within the species. Further research is needed to explore the causes that give rise to the subtle changes detected in the wing morphology of *Phulia nymphula*, to evaluate the presence of phenotypic plasticity in the studied morphological trait, and to understand how the species fitness and population dynamics are affected under the unique conditions of the Chilean Altiplano.

## Figures and Tables

**Table 1:** Permutation test among 6 localities of the Northen Altiplano (Caquena, Sorapata Lake, Chungará, Casiri Macho Lake, Surire Salt Flat, and Visviri) using Mahalanobis distances. *p*-values from permutation tests (10,000 permutation rounds).

Distances/*p*-Values	Caquena	Chungará	Casiri Macho Lake	Sorapata Lake	Surire Salt Flat
Chungará	7.6499 0.0424				
Casiri Macho Lake	7.3830 <0.0001	8.6599 0.0058			
Sorapata Lake	6.7149 <0.0001	9.0319 0.0036	2.4913 0.0084		
Surire Salt Flat	5.7120 <0.0001	4.6412 0.0368	5.6889 <0.0001	5.6086 0.0505	
Visviri	7.2949 <0.0291	5.2953 0.3342	7.9680 0.0050	8.2105 0.0768	4.6450 0.0474

**Table 2:** Wind speed by location: average and maximum values (m/s). Wind speed categories, elevation, and basins/sub-basins.

Location (n)	General Average Speed *	Maximum Speed **	Category	Elevation (m.a.s.l.)	Basin/Sub-Basin ***
Visviri (2)	4.47	17.47	LOW (<20)	4084	Putani River
Casiri Macho Lake (12)	6.60	30.47	HIGH (>25)	4844	Caquena River
Sorapata Lake (22)	7.10	28.54	HIGH (>25)	5200	Caquena River
Caquena (5)	4.00	17.56	LOW (<20)	4401	Caquena River
Chungará (2)	5.80	24.23	MEDIUM (20–25)	4568	Chungará Lake
Surire Salt Flat (33)	4.90	20.61	MEDIUM (20–25)	4268	Surire Salt Flat

* RECON Model 1980–2017; ** at 5.5 m (6:00–18:00), 180,440 records; *** [49,50].

## Data Availability

Data will be made available by personal request to the corresponding author.

## Supplementary Materials

The following supporting information can be downloaded at: https://www.mdpi.com/article/10.3390/ani14192758/s1, Figure S1: Daily wind speed over three consecutive years: 2015, 2016, and 2017 (RECON Model 1980–2017); Figure S2: Daily wind speed in austral winter and spring (RECON Model 1980–2017); Table S1: *p*-values in paired comparisons, PERMANOVA, 9999 permutations *p* < 0.01; Bonferroni correction.
