# Supplementary material for: Unveiling the Wing Shape Variation in Northern Altiplano Ecosystems: The Example of the Butterfly Phulia nymphula Using Geometric Morphometrics
Source: Animals (Basel). 2024 Sep 24;14(19):2758. doi: 10.3390/ani14192758 (PMC11476329; doi:10.3390/ani14192758)
Supplement: Supplementary file 1 [file animals-14-02758-s001.zip › animals-3152854-supplementary.pdf]

Table S1: p-values in paired comparisons, PERMANOVA, 9999 permutations p<0.01; Bonferroni correction

	LOW (<20)	MEDIUM (20-25)
MEDIUM (20-25)	0.0051	
HIGH (>25)	0.0003	0.0003

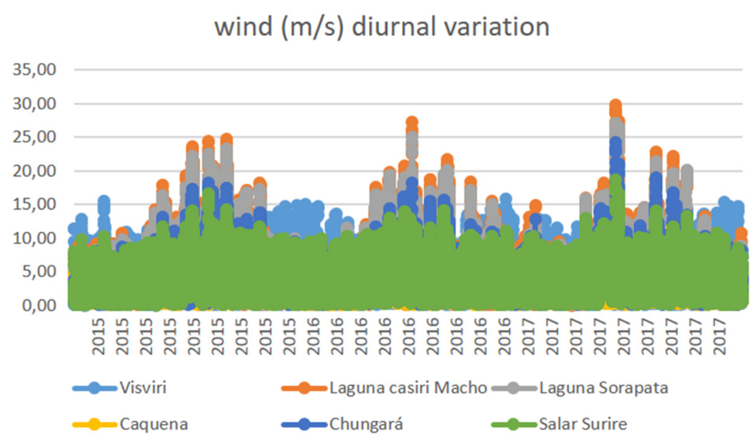

Figure S1: Daily wind speed over three consecutive years: 2015, 2016, and 2017 (RECON Model 1980-2017).

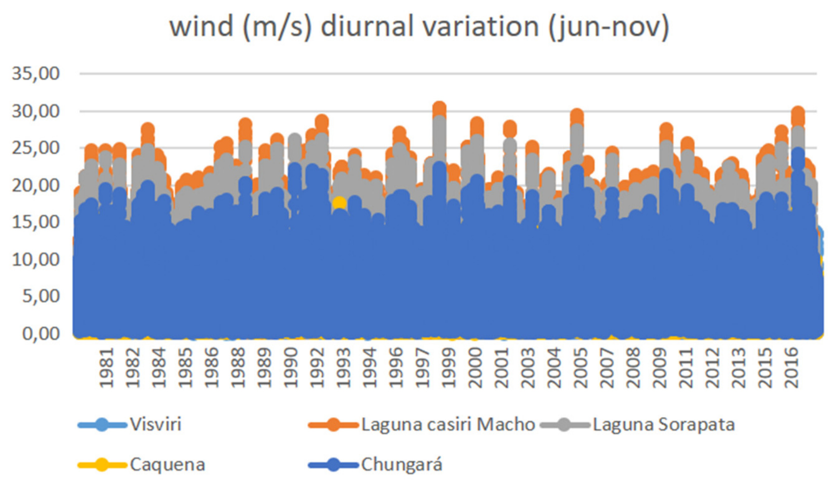

Figure S2: Daily wind speed in austral winter and spring (RECON Model 1980-2017).
